# Supplementary figures and images for: Efficacy of the Feliway® Classic Diffuser in reducing undesirable scratching in cats: A randomised, triple-blind, placebo-controlled study
Source: PLoS One. 2023 Oct 18;18(10):e0292188. doi: 10.1371/journal.pone.0292188 (PMC10584138; doi:10.1371/journal.pone.0292188)

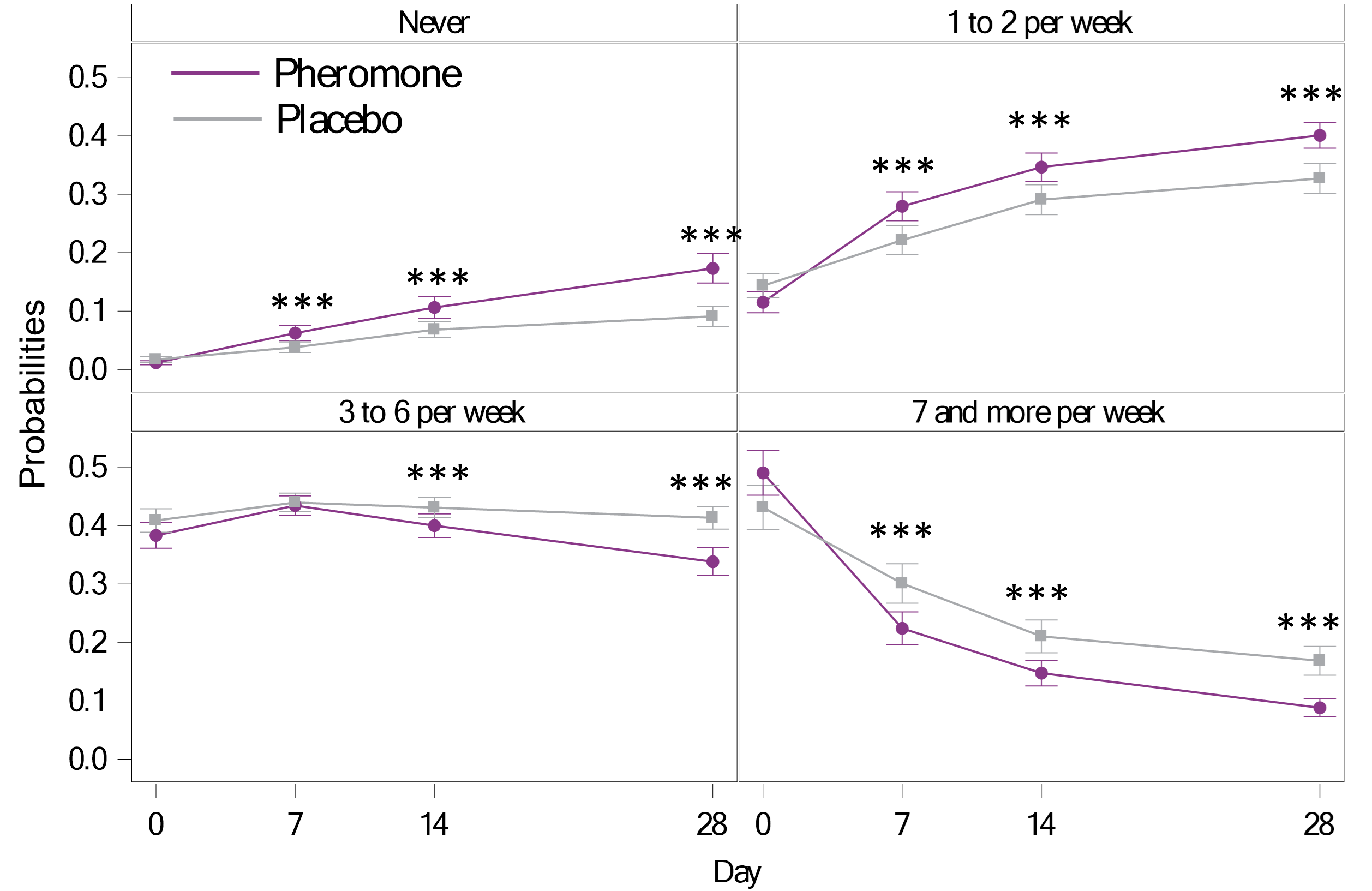

Supplement: S1 Fig — (TIF) [file pone.0292188.s001.tif]

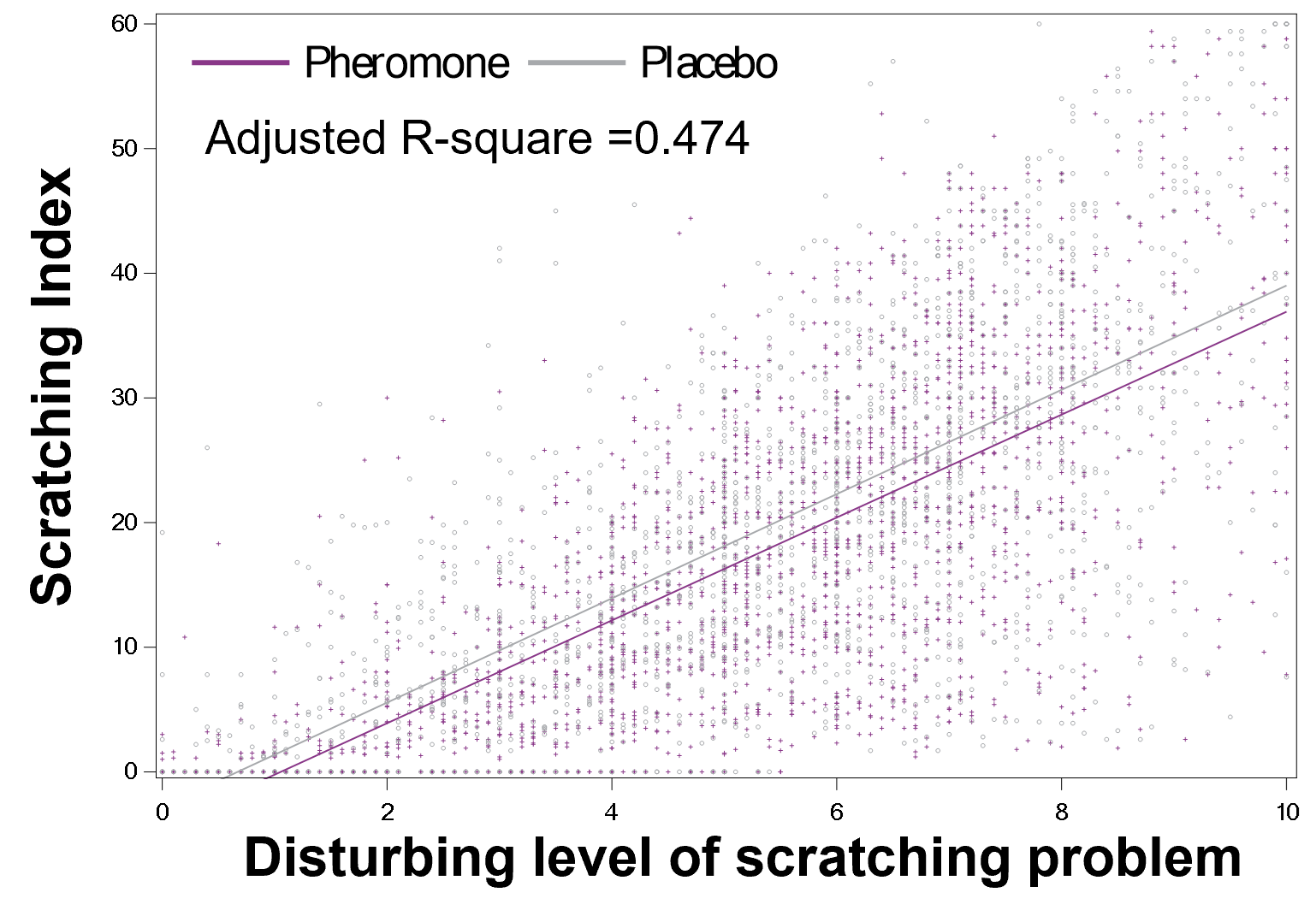

Supplement: S2 Fig — (TIF) [file pone.0292188.s002.tif]

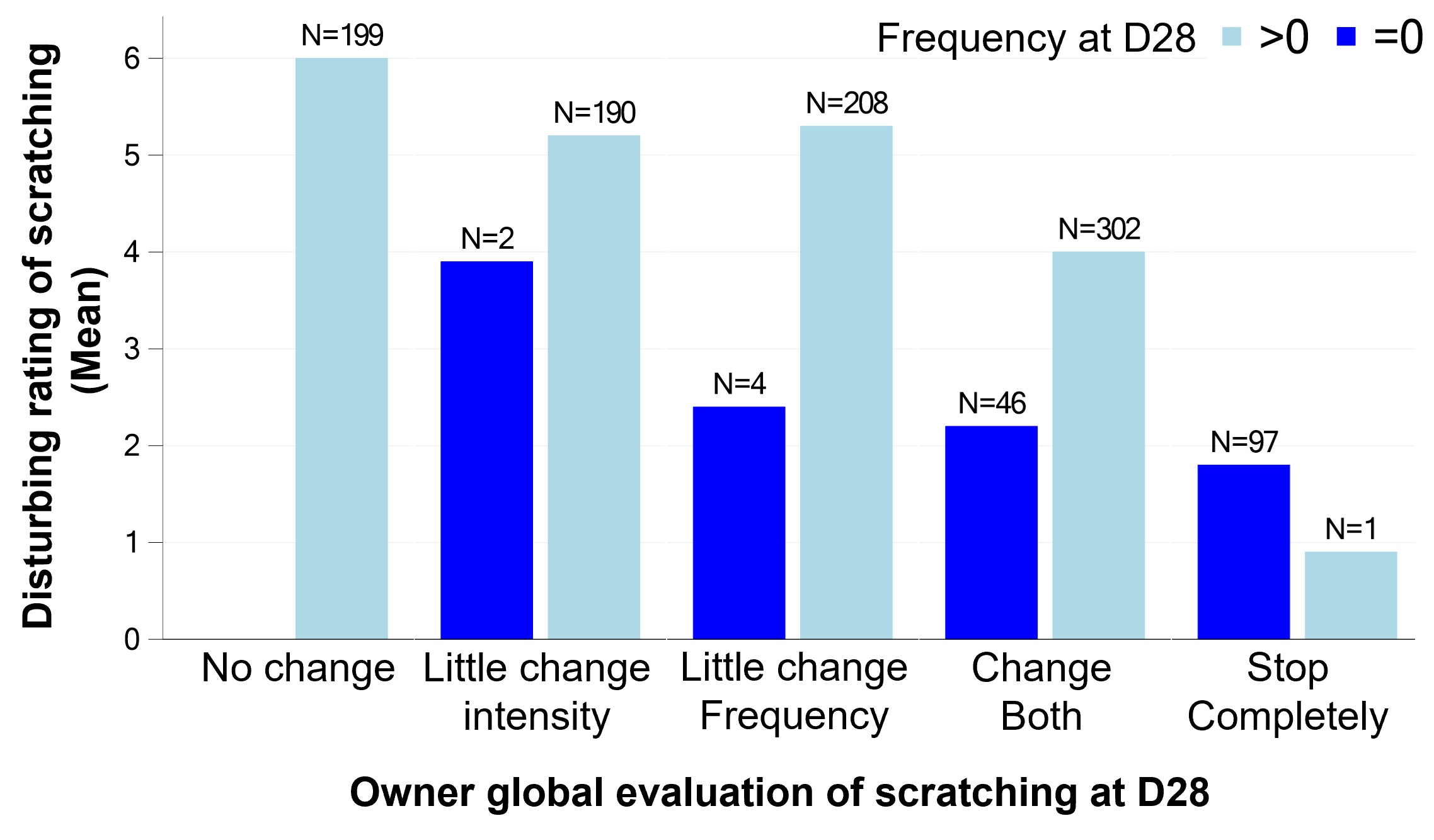

Supplement: S3 Fig — (TIF) [file pone.0292188.s003.tif]

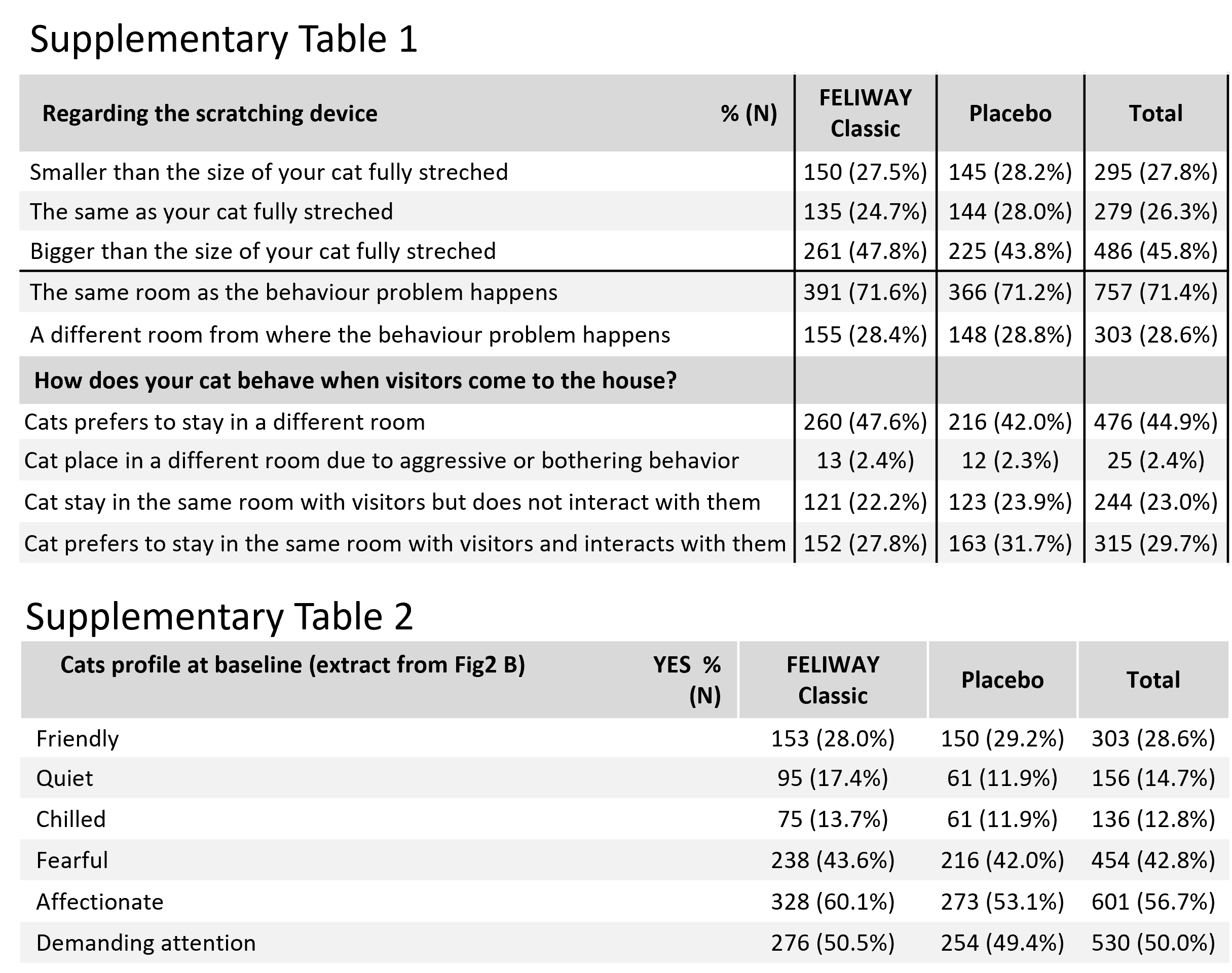

Supplement: S3 File — Cats profile at baseline (extract from Fig 2B). (TIF) [file pone.0292188.s006.tif]
