# Supplementary material for: Efficacy of the Feliway® Classic Diffuser in reducing undesirable scratching in cats: A randomised, triple-blind, placebo-controlled study
Source: PLoS One. 2023 Oct 18;18(10):e0292188. doi: 10.1371/journal.pone.0292188 (PMC10584138; doi:10.1371/journal.pone.0292188)
Supplement: S1 File — (PDF) [file pone.0292188.s004.pdf]

# Data

| Gender and neutered status | | FELIWAY Classic (N=546) | Placebo (N=514) |
|---|---|---|---|
| A male not neutered | n (%) | 27 (4.9%) | 29 (5.6%) |
| A female not spayed | | 31 (5.7%) | 28 (5.4%) |
| A male neutered | | 228 (41.8%) | 232 (45.1%) |
| A female spayed | | 260 (47.6%) | 225 (43.8%) |

| | | | FELIWAY Classic (N=546) | Placebo (N=514) | FELIWAY Classic (N=546) | Placebo (N=514) | | FELIWAY Classic (N=546) | Placebo (N=514) |
|---|---|---|---|---|---|---|---|---|---|
| | **Statistics** | **Days** | **Value** | | **Absolute change from D0** | | **Statistics** | **Absolute change from D0** | |
| **Intensity** | *Mean(SD)* | Day 0 | 6.4 (1.5) | 6.3 (1.5) | | | *Mean (95% CI) Fig, 3 B* | 0 ( 0; 0) | 0 ( 0; 0) |
| | | Day 7 | 5.3 (2.1) | 5.5 (2.1) | -1.2 (1.9) | -0.8 (1.7) | | -1.2 ( -1.3; -1) | -0.8 ( -0.9; -0.7) |
| | | Day 14 | 4.4 (2.2) | 4.6 (2.3) | -2.0 (2.1) | -1.6 (2.0) | | -2 ( -2.1; -1.9) | -1.6 ( -1.8; -1.5) |
| | | Day 28 | 3.7 (2.3) | 4.3 (2.4) | -2.7 (2.2) | -2.0 (2.2) | | -2.7 ( -2.9; -2.6) | -2 ( -2.1; -1.8) |
| **Frequency** | *Median (95% CI) Fig, 3 A* | Day 0 | 4 ( 4; 4) | 4 ( 4; 4) | | | | | |
| | | Day 7 | 3 ( 3; 4) | 4 ( 4; 4) | | | | | |
| | | Day 14 | 3 ( 2; 3) | 3 ( 3; 3) | | | | | |
| | | Day 28 | 2 ( 2; 2) | 3 ( 3; 3) | | | | | |
| **Index** | *Mean(SD)* | Day 0 | 28.3 (11.4) | 26.8 (12.0) | | | *Mean (95% CI) Fig, 3 C* | 0 ( 0; 0) | 0 ( 0; 0) |
| | | Day 7 | 18.7 (11.9) | 21.7 (13.6) | -9.6 (10.8) | -5.0 (9.6) | | -9.6 ( -10.3; -8.8) | -5 ( -5.7; -4.3) |
| | | Day 14 | 14.4 (11.6) | 17.3 (13.4) | -13.9 (11.5) | -9.5 (10.1) | | -13.9 ( -14.7; -13.1) | -9.5 ( -10.2; -8.7) |
| | | Day 28 | 10.8 (10.7) | 15.7 (13.5) | -17.5 (11.7) | -11.0 (10.4) | | -17.5 ( -18.3; -16.7) | -11 ( -11.8; -10.3) |
| **Disturbing** | *Mean(SD)* | Day 0 | 6.6 (1.8) | 6.4 (1.8) | | | *Mean (95% CI) Fig, 4 A* | 0 ( 0; 0) | 0 ( 0; 0) |
| | | Day 7 | 5.7 (1.9) | 6.0 (2.1) | -0.9 (1.7) | -0.5 (1.6) | | -0.9 ( -1; -0.7) | -0.5 ( -0.6; -0.3) |
| | | Day 14 | 5.0 (2.1) | 5.1 (2.3) | -1.6 (1.9) | -1.3 (1.9) | | -1.6 ( -1.7; -1.5) | -1.3 ( -1.5; -1.2) |
| | | Day 28 | 4.4 (2.3) | 4.7 (2.4) | -2.2 (2.1) | -1.7 (2.1) | | -2.2 ( -2.4; -2.1) | -1.7 ( -1.9; -1.5) |

| **Overall, for the last 28 days do you think the scratching problem** | | | | |
|---|---|---|---|---|
| | **Statistics** | **Days** | **FELIWAY Classic (N=546)** | **Placebo (N=514)** |
| Is worse – the cat scratches with a higher frequency and with the higher intensity | *% (n)* | Day 28 | 0.2% (1) | 0.6% (3) |
| Is worse – the cat started scratching also in other places | | | 0.4% (2) | 1.0% (5) |
| Didn’t change at all - the cat scratches with the same frequency and with the same intensity | | | 10.6% (58) | 27.4% (141) |
| Changed a little – the cat scratches with the same frequency but with lower intensity | | | 17.8% (97) | 18.5% (95) |
| Changed a little – the cat scratches with a lower frequency but with the same intensity | | | 22.5% (123) | 17.3% (89) |
| Changed a lot – the cat scratches with less frequency and less intensity | | | 37.4% (204) | 28.0% (144) |
| Changed completely – the cat stopped scratching | | | 11.2% (61) | 7.2% (37) |
