# Supplementary material for: Efficacy of the Feliway® Classic Diffuser in reducing undesirable scratching in cats: A randomised, triple-blind, placebo-controlled study
Source: PLoS One. 2023 Oct 18;18(10):e0292188. doi: 10.1371/journal.pone.0292188 (PMC10584138; doi:10.1371/journal.pone.0292188)
Supplement: S2 File — (PDF) [file pone.0292188.s005.pdf]

Weekly questionnaire (Questionnaire Hebdomadaire)

Since the last questionnaire…

1. Have you kept the diffuser plugged into the outlet 24 hours a day?
   1- Yes
   2- No

<u>If code 2</u>

2. Did you unplug the diffuser?
   1- Less than 12h in total
   2- Definitely

3. For what reason did you unplug the diffuser?

4. A.Please could you choose the set of picture that reflect better the seven past day of your cat ?

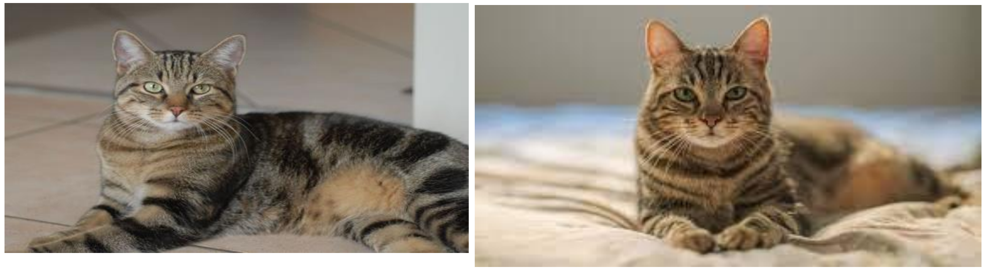

a

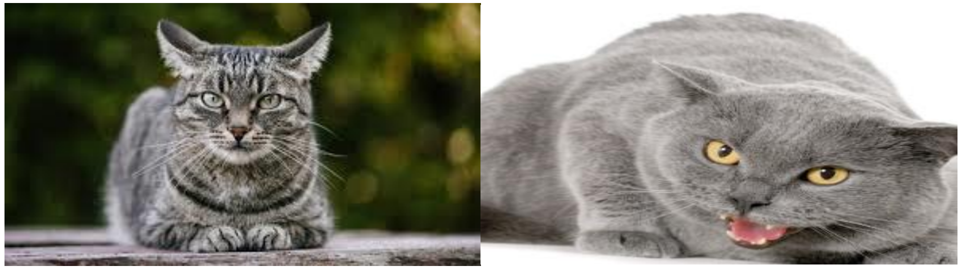

b

Depuis le dernier questionnaire…

1. Avez-vous laissé le diffuseur branché sur la prise 24 heures sur 24 ?
   1- Oui
   2- Non

<u>Si le code 2</u>

2. Avez-vous débranché le diffuseur ?
   1- Moins de 12h au total
   2- Définitivement

3. Pour quelle raison avez-vous débranché le diffuseur ?

4. A. Pourriez-vous choisir le duo de photos qui reflète le mieux les sept derniers jours de votre chat ?

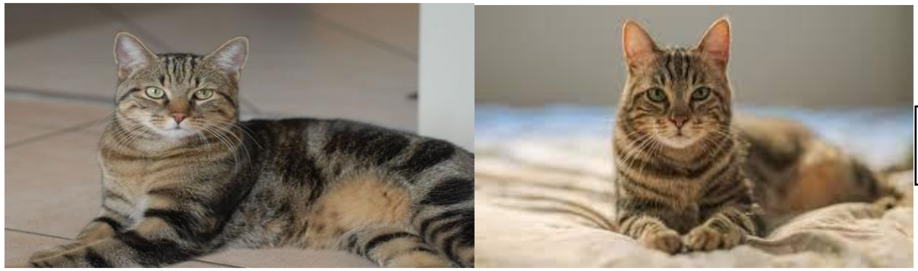

a

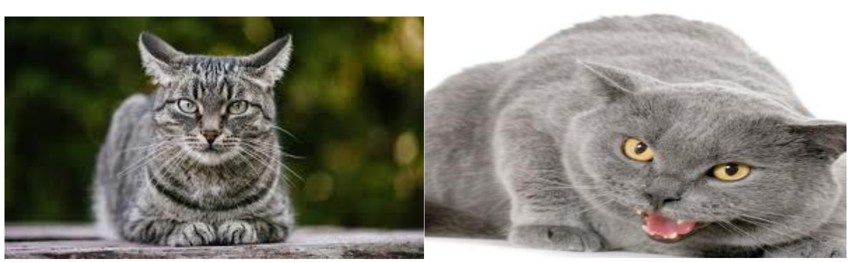

b

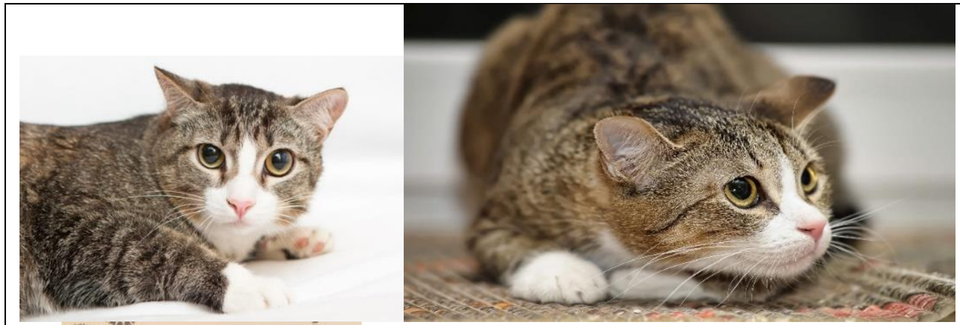

c

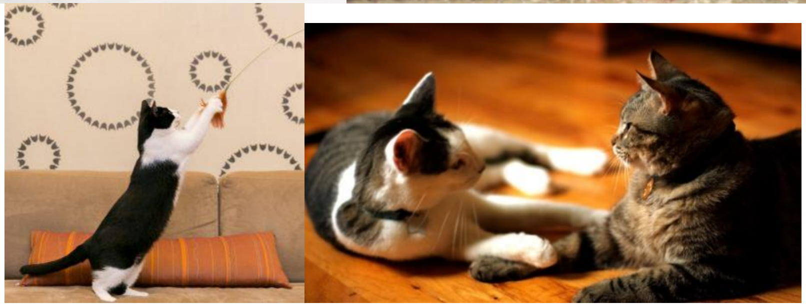

d

5. B. To what extent did you consider this scratching problem is disturbing for you and your household? (from 0 “it is not disturbing at all” to 10 “it is extremely disturbing”) *(PROG. VAS from 0 to 10, from 0,1 to 0,1)*

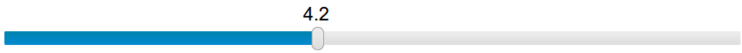

6. C.In the last 7 days, how often have you noticed your cat has made inappropriate scratches (Scratching indoors on vertical surfaces other than the cat tree/scratching post/other scratching device you provide him e.g. sofa, furniture, curtains, door frames...)? The inappropriate scratching could be directly observed or deduced from new damage observed.

6. Every day, more than twice a day
5. Every day, once or twice a day
4. Almost every day
3. Every other day
2. twice a week
1. Once a week

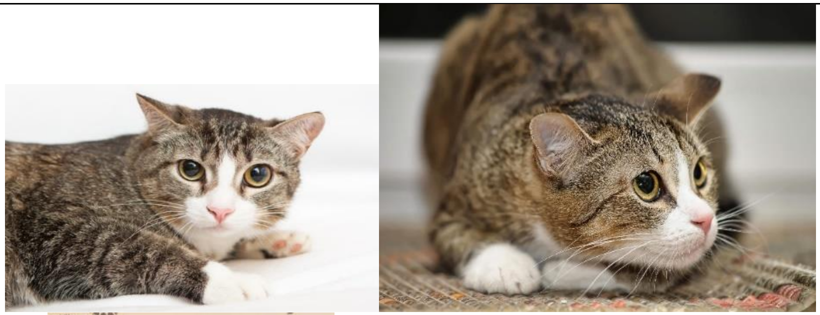

c

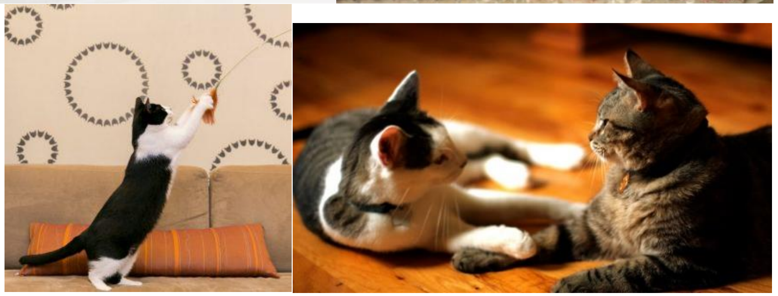

d

5. B. Dans quelle mesure considérez-vous que ce problème de griffade est dérangeant pour vous et votre foyer ? (de 0 "ce n'est pas du tout gênant" à 10 "c'est extrêmement gênant") *(VAS. de 0 à 10, de 0,1 à 0,1)*

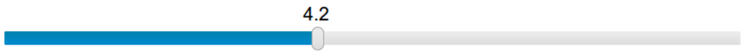

6. C. Au cours des 7 derniers jours, combien de fois avez-vous remarqué que votre chat a fait des griffades inappropriées (griffades à l'intérieur sur des surfaces verticales autres que l'arbre à chat/ griffoir/dispositif de grattage que vous lui fournissez, par exemple canapé, meubles, rideaux, cadres de porte...) ? Les griffades inappropriées peut être observées directement ou déduit de nouveaux dommages observés.

6. Tous les jours, plus de deux fois par jour
5. Tous les jours, une ou deux fois par jour
4. Presque tous les jours
3. Tous les deux jours
2. deux fois par semaine

0. Never

STOP if code 1or 0

7. D_If you have in mind the exact number of times your cat has performed this behavior, please indicate it

/ __________________/ times per week

8. E_(If different from 0 to frequency) Still regarding [name completed in Q1 BASELINE], disregarding frequency, what do you think is the current average intensity of his scratching? Please place the cursor on this scale to describe the current intensity of this bad habit *(VAS PROG. of 1 to 10, 0.1 to 0.1)*. the intensity can be evaluate with the duration of the scratch and/or the severity of the damage observed.

Extremely low intensity Extremely high intensity

9. Did your cat change the place preferred for the problem scratching?
   1. No
   2. Yes

Is yes please state from where to where :_________

10. F_ In the past 7 days, did you where present at home awake at least 4 hours per day and at least 5 days ?

11. H_In the past 7 days, has your cat undergone changes in environment or habits such has:
    1. Change in the environment (e.g. moving to a new house / new pet / strangers / major change of furniture ...). Please specify
    2. General change in household routine (complete change in the schedules of the owners and/or the cat), please specify
    3. No change

1. Une fois par semaine
0. Jamais

STOP si code 1ou 0

7. D_Si vous avez en tête le nombre exact de fois où votre chat a eu ce comportement, veuillez l'indiquer.

/ __________________/ fois par semaine

8. E_(Si différent de 0 à la fréquence) Toujours en ce qui concerne [nom rempli dans Q1 BASELINE], sans tenir compte de la fréquence, quelle est, selon vous, l'intensité moyenne actuelle de ses griffades ? Veuillez placer le curseur sur cette échelle pour décrire l'intensité actuelle de cette mauvaise habitude *(PROG. VAS de 1 à 10, 0.1 à 0.1)*. *L'*intensité peut être évaluée en fonction de la durée des griffades et/ou de l'étendue des dommages observés.

Intensité extrêmement faible Intensité extrêmement élevée

9. Votre chat a-t-il changé l'endroit préféré pour ses griffades problématiques ?
   1. Non
   2. Oui

Si oui, veuillez préciser de quel endroit à quel endroit :_________

10. F_ Au cours des 7 derniers jours, avez-vous été présent à la maison, éveillé, au moins 4 heures par jour et au moins 5 jours ?

11. H_Au cours des 7 derniers jours, votre chat a-t-il subi des changements d'environnement ou d'habitudes tels que :
    1. Changement d'environnement (par exemple, déménagement dans une nouvelle maison / nouvel animal de compagnie / étrangers / changement important de mobilier ...). Veuillez préciser
    2. Changement général dans la routine du foyer (changement complet des horaires des propriétaires et/ou du chat), veuillez préciser

12. I_ Has your cat received any treatment in the past 7 days?
    1. Yes
    2. No

If yes

13. J_Which one?

14. Since the last questionnaire, has/have your cat or pet(s) been bothered / affected by the product?

    1- Yes
    2- No

    If code 1

15. Please detail as much as possible the discomfort(s) felt (a: which pet, b: what date(s) it happened, c : how long, d : how many times, e : detailed description of the discomfort(s)

16. Other comments / remarks

Thank you for taking the time to complete this questionnaire. You will be asked to answer the following questionnaire in 7 days. If in the meantime for any reason you need to unplug the diffuser, please contact the agency.

3. Pas de changement

12. I_ Votre chat a-t-il reçu un traitement au cours des 7 derniers jours ?
    1. Oui
    2. Non

Si oui

13. J_Lequel ?

14. Depuis le dernier questionnaire, votre chat ou l'un de vos animaux de compagnie ont-ils été gênés/affectés par le produit ?

    1- Oui
    2- Non

    Si le code 1

15. Veuillez détailler autant que possible le(s) gênes(s) ressentie(s) (a : quel animal, b : quelle(s) date(s) cela s'est produit, c : combien de temps, d : combien de fois, e : description détaillée de la (des) gêne(s)

16. Autres commentaires / remarques

Merci d'avoir pris le temps de répondre à ce questionnaire. Vous serez invité à répondre au questionnaire suivant dans 7 jours. Si entre-temps, pour une raison quelconque, vous devez débrancher le diffuseur, veuillez contacter l'agence.
