# Supplementary material for: Efficacy of the Feliway® Classic Diffuser in reducing undesirable scratching in cats: A randomised, triple-blind, placebo-controlled study
Source: PLoS One. 2023 Oct 18;18(10):e0292188. doi: 10.1371/journal.pone.0292188 (PMC10584138; doi:10.1371/journal.pone.0292188)
Supplement: Attachment — Submitted filename: Cover Letter for plosone re-submission.docx [file pone.0292188.s007.docx]

 Xavier De Jaeger

Ceva Santé Animale - France

10 Avenue de la Ballastière,

Clinical department

33500 Libourne

July 16^th^ 2023

Dear Dr. Brundage,

We sincerely appreciate your response dated 2 June 2023, inviting us to revise and resubmit our manuscript initially titled "The impact of the use of FELIWAY® Classic Diffuser in reducing undesirable scratching in cats." We are pleased to submit the latest version of our manuscript, now titled "Efficacy of the Feliway® Classic Diffuser in reducing undesirable scratching in cats: a randomized, triple-blind, placebo-controlled study," as per the suggestion of the reviewer.

We would like to express our gratitude to the reviewers for their valuable feedback and constructive comments, which have greatly contributed to the enhancement of our work.

We have diligently addressed the reviewer comments and incorporated their suggestions into the revised manuscript. To facilitate the review process, we have uploaded the revised manuscript alongside this letter. The modifications made in response to the reviewer comments are clearly marked in blue in the following table, allowing for easy identification.

We are confident that the revisions made have significantly improved the quality and clarity of our manuscript. We sincerely hope that the revised version will meet the publication criteria of PLOS ONE and be considered for publication.

Thank you once again for your time and consideration. We eagerly await your decision.

Sincerely,

Xavier De Jaeger

Edits requested on your submission PONE-D-23-09288R1

1) The Competing Interests statement has been updated in the manuscript line 421 as follow:

LM, SE and XDJ are employees of Ceva Santé Animale.

JSP, YS, GGP authors received fees from Ceva Santé Animale for their contribution on the study design, interpretation of results and manuscript writing. This does not alter our adherence to PLOS ONE policies on sharing data and materials.

2) Indeed, they was an error since employee of Ceva Sané animale was involved in the study. Here the statement that can be amended:
This study was funded by Ceva Santé Animale who provided the diffusers and was implicated in all the step of this clinical trial.

3) I am sorry, but I did not find which figure you are referring. I was not able to find it, here all the supplementary figures are with the file type “Supporting Information” as you can see below:

4) The financial disclosure has been removed from the manuscript

5) This has been added Line 112

General comments:

1) We hope that the modification made on the revised manuscript will cover the reviewer expectation about the data and the conclusion.
2) We added several information concerning the statistic to be clarify what was made
3) In the supportive information the minimal data set were available as describe in the PLOS data policy. The values used to build graphs are fully available and the questionnaire used are also available, as mentioned in the policy we do not need submit the raw data collected.
4) The modification made on the revised manuscript should help the manuscript to be more intelligible.
5) See our response to the reviewers.

Reviewer #1

| **No.** | **Comment/Suggestion** | **Response** | **Line** |
| --- | --- | --- | --- |
| 1 | I would suggest making the title more specific; perhaps you could change from “The impact of the use of FELIWAY® Classic Diffuser in reducing undesirable scratching in cats” to: “Efficacy of the Feliway ® Classic Diffuser in reducing undesirable scratching in cats: a randomised, triple-blind, placebo-controlled study” | We changed the title according to the suggestion of the reviewer. | Line 1 |
| 2 | You have a nice abstract, but I would suggest mentioning a little bit as introduction the scratching behaviour (if word count allows) – | We added some introduction regarding the scratching behaviour. | Line 15-17 |
| 3 | The introduction is nice, clear, and well-written. It has a nice flow and clearly states the aims of the study.  Perhaps a little bit of extra detail on the conclusion may be useful | More details were added | Line 406-410 |
| 4 | To manage scratching on furniture I would like to know from a general overview of the recent and current studies if it were better to use F3, FIS or both in reducing scratching in terms of efficacy and costs | We can say that both products seem to reduce the scratching the environment as stated in line 292, however, we believe that in order to have comparison of the efficacy the two products or even the combination of both we would need studies that were similar (triple-blinded, placebo controlled) or a separate study where these products would be used in the same condition and unfortunately this is not the case so we believe this comparison should not be done in a published study as we don’t have the means to make such comparisons. Regarding the costs it is also dependent from country to country so is it not possible for us to make comparisons on that matter. |  |
| 5 | The reference 3 is incomplete | This has been Corrected | Line 441 |

Reviewer #2

| **No.** | **Comment/Suggestion** | **Response** | **Line** |
| --- | --- | --- | --- |
| 1 | Line 16: consider with instead of ‘that included’ especially considering line 18 also uses the word included | We have changed this in the revised manuscript | Line 21 |
| 2 | Line 25- what does ‘one category’ mean? The reader doesn’t know enough at this point to understand that | For clarity we remove the term category. | Line 30 |
| 3 | Line26: nice to have p values, please explicitly state which group had lower scratching at these days with is the key finding | The p value referred to the difference in percentage cited just before we added for clarity “of the cats” |  |
| 4 | Line 30: include the word diffuser after Classic | We have changed this in the revised manuscript | Line 35 |
| 5 | Line 35- do we have a citation to support this sentence? | Added more references besides previous #1 and #2. These new ones also include the sentence in line 35 of the original manuscript so they were added after this sentence. | Line 41 |
| 6 | Line 37- ‘will’ is unnecessary | This has been corrected | Line 42 |
| 7 | Line 40-41, the sentence starting with ‘The use of scratching as a marking signal is expected’ seems out of place in this paragraph and may be more relevant later in the introduction. Consider shortening by removing ‘that’. Please also add a citation | Changed according to suggestion | Line 46 |
| 8 | Line 51- citation 7 is really only relevant to onychectomy, please provide citations for the other recommenda | Citations added | Line 55 |
| 9 | Line 51-56- this is awkward to have lumped into one sentence for the reader, please consider splitting and reworking (the phrase ‘a procedure’ is also unnecessary, it’s not clear what the different populations of cats are here). | Changed according to suggestion | Line 55-57 |
| 10 | Line 61- this would be a good place to introduce scratcher preferences, needs | Changed according to suggestion | Line 65-71 |
| 11 | Line 63- ‘being’ is unnecessary | This has been corrected | Line 73 |
| 12 | Line 63-67 is an awkwardly long sentence | This has been changed in order to be more succinct | Line 75-76 |
| 13 | Lines 88-92 this seems like the sort of calculation that might belong under statistical analysis | This was moved to the statistical part | Line 196-173 |
| 14 | Also what kind of regression model? | A linear model (change=treatment) | Line 196 |
| 15 | There should be more information about recruiting here- what kind of panel of cat caregivers, how were they recruited other than emailed (were they sent the whole survey? Or asked to sign up?) | some clarifications were made | line 97-99 and line 109-112. |
| 16 | Line 110-112- please consider starting the paragraph with this sentence or put it in the design section. Please also include the window of opportunity allowed for each time point- 24 hours in other side, only on the day of? 3 days after the email notification? | This was added | line 111-112 |
| 17 | Line 117: consider lumping all of the human participant information together (age, consent). Then you can separate out all the cat inclusion and exclusion criteria | This information was added |  |
| 18 | Line 120: please define 2 per week- 2 bouts, 2 minutes, 2 hours? | the scale is detailed the line after | Line 139-141 |
| 19 | Line 121: observed (aka the client saw the cat doing the damage) or found (during or after?) | both it is specify: ” directly observed or deduced from new damage” | Line 138 |
| 20 | Line 123- likert scales can be treated statistically as semi-quantitative scales, but this should be supported with a citation. Also consider changing ‘every day’ to ‘daily’ in the text for brevity and ease of reading, even if that is not what the survey said. | The reference has been added (Suillivan GM and Artino AR 2013, DIO: [10.4300/JGME-5-4-18](https://doi.org/10.4300%2FJGME-5-4-18)). Concerning the change ‘every day’ to ‘daily’, we rather keep the exact mention that has been used and provided to the caregivers. | Line 141 |
| 21 | Line 125-126 Were examples of intensity provided or was it entirely arbitrary? | No example was provided to owners |  |
| 22 | Line 128- tense, please switch to past tense | This has been corrected | Line 145 |
| 23 | Line 131- “from these two criteria’ is redundant | This has been removed | Line 147 |
| 24 | Line 133: “in addition to this is unnecessary”. Please confirm the clients were asked to rate disturbing on day 0 here, as only day 28 is mentioned. Consider a table or using different demarcation system than parentheticals inside parentheticals. | This has been changed | Line 149 |
| 25 | Somewhere in this section, the temperament/personality questions need to be introduced instead of in the statistical analysis. | Details added on the first paragraph. | Line 115-120 |
| 26 | EXCLUSION CRITERIA- could consider having this only be a table and earlier in the method section | We believe this section of the materials and methods needs to be after the “data collection” (explanation about questionnaires) as some of the participants were only excluded after the evaluation the questionnaires. For example, some demographic characteristics (of the owner, the cat and the environment) such as age, number of pets, changes in the environment are criteria that may implicate exclusion. | Line 166 Table 1 |
| 27 | Please define “Major environmental change” is that a new piece of furniture, a move, someone leaving the household, all of the above? | Some examples added to the table. | Line 166 Table 1 |
| 28 | Is there a citation/justification for needing the client to observe 4/hrs a day? | No there is no citation we try to choose an amount of time that seems feasible for someone working and sufficient to observe the cat. |  |
| 29 | You need to include having a data point missing as exclusion criteria since it later showed up as a reason for exclusion (table 3) | This has been added | Line 166 Table 1 |
| 30 | Line 150-152- this sentence does not belong in the statistical analysis section, that’s data collection. The sentence on the Global Index Score is redundant to the previous section. | Indeed this is redundant but since this is an important point to understand what has been done we would like to keep it. | Line 175-177 |
| 31 | Line 153-155- you state it’s not appropriate to report significance, but then later do so, and most scientific readers will want to know anyway if the two groups were different, even if we know it was just due to chance, because it changes our interpretation of the comparisons later. | Indeed, some scientific readers are used to this, but this does not make sense statistically speaking. This will not change the interpretation of the comparison later since the baseline values are added in the models to adjust the results on it. This will not bias or change the conclusions. |  |
| 32 | Line 161- please justify why you wanted to limited the number of categories as you lose data and variability when doing this- small cells? | This will increase the number of cats in each category and will help the model to be more accurate. This also eases the interpretation since we will compare less modalities together. |  |
| 33 | Line 165- evolution is not an accurate term here, we are not watching changes over generations, please revise all instances of this verb. | We meant to use it as “a gradual process of change” and not as the biological sense of evolution. This has been changed. | Line 192 |
| 34 | Line 166- why was Chi squared sused for the Global Index score? It was the closest to truly continuous variable you had. It’s statistically appropriate to do so (not violating assumptions) but not a choice many would make and would love to see some discussion on this | The parameter used here is a reclassification of the Global index score in 2 categories. It is not anymore a quantitative but a qualitative parameter. It's classical to use a Chi squared test to compare 2x2 crossing. The sentence was reformulated to clarify. | Line 192 |
| 35 | Line 167- this sentence is unclear because of ‘the same analyses were performed’. If you mean as each other, you can remove that phrase and join ‘for intensity and global index score’ a XXXXX was used. However, in the sentence just above it, you state that Chi squared was used. | The sentence was reformulated to clarify | Line 194 |
| 36 | Line169- what kind of longitudinal model (linear, survival?) and how was it built? ‘ per behaviour observation’ could be shortened to ‘by’ to make the sentence easier to read. | The evolution (change from baseline) of intensity of scratching and Global Index Score was analyzed using a longitudinal mixed model with repeated measures.  Visit day and baseline value were fixed effects as well as the treatment group.  Cat age, lifestyle, presence of behavior problem, emotional health, time spent at home, other cats at home, other pets at home, owner’s family situation, location of the scratching and number of the scratching post were tested as fixed effect and kept if necessary (determined by Akaike’s information criterion) (MIXED procedure of SAS).  The animal was tested as a random effect.  These precisions have been added in the reviewed version. | Line 193-201 |
| 37 | Line 173- do you mean generalized least square means? | Yes. It has been added in the reviewed version. | Line 193-201 |
| 38 | Line 183- ‘for different reasons’ is unnecessary just refer to the table | Changed according to suggested. | Line 2015 |
| 39 | Line 191- this is where you contradict lines 153-155 | Here, we just compare the value without any statistical test, line 153-155 we claim that performing statistical test is not correct, but we can have a look on the distribution without using stat. | Line 222 |
| 40 | Line 194- first time the phrase ‘selection phase’ is mentioned- please define in methods section and in diagram | The selection questionnaire is already in the diagram. The term ‘phase” has been removed | Line 226 |
| 41 | Line 197- first time the cat personality profile is mentioned other than abstract, include in methods. Please also expand on this section is this a validated scale? | Information added on the methos section. | Line 115-120 |
| 42 | Line 204: Figure 2 caption- this is not particularly clear if you are displaying frequency and intensity of scratching, label it that instead of ‘data’ Item c- caregiver involvement is an entirely new variable that did not appear anywhere in the method section | C) is not a variable this is just the title of what we intend to show with this graph. Here we are just displaying the frequency at two different time points to see if a change is observed. |  |
| 43 | Line 212- I think you mean “non-continuous”? please remove unnecessary words for clarity- the, parameter, of the scratching frequency. In, not on. | Yes, it has been modified in the reviewed version. | Line 243 |
| 44 | Line 213- which quantitative test? And why ‘but’ the ordinal regression model was used? This should also be discussed in the statistical analysis section. | A Wilcoxon test. ‘But’ was replaced by ‘and’. The sentence was rephrased. Information was added in the statistical analysis section. | Line 244-245 |
| 45 | Line 215-221: Your figure has no asterisks suggesting visually that none of these comparisons you are listing were statistically significantly different.. no p values are reported in this section. | Added in the new version | S1 Fig |
| 46 | Line 221-23- this type of evaluative sentence goes in the discussion, numbers only for the results | This is just what we can extract from the results, not really a discussion of the results. In consequence we thought that it worth to keep it here. | Line 253-255 |
| 47 | Lines 224-228: please tell us a little bit about the direction of these differences and interactions | It was added in the new version. | Line 255-260 |
| 48 | Line 229: shorten to ‘ The Global Score’ is presented in Figure 3C. | This has been changed | Line 261 |
| 49 | Line 230- again which kind of mixed model? With what variables as repeated/between/predictors? This information isn’t in the supplementary material either | Details added in the statistical analysis section | Line 262 |
| 50 | Line 231- would stick with calling this variable ‘day’ vs study day, please also here expand a little on the interaction. Most models I’ve seen presented will also provide table with the intercepts, effect size and variables. | Concerning “day” this has been changed. About the details on the model since none of the covariable interact significantly with the product application we did not provide all the details to not overload the paper. In the statistical methodology it was mentioned that “the interactions between the covariables and the treatment group were tested and kept if significant to fit the best model” we added a precision in the result part | Line 206-209  And 261-263 |
| 51 | Line 237- typically the asterisk and significance goes in the caption vs the figure title | Sorry but here we are not sure to understand what was asked. The asterisk are after the tittle in the caption. | Line 268 |
| 52 | Line 240- please list the variables because it’s not clear from your materials and methods and/or say ‘no other significant interaction was found | We changed to “(see table 2)” because it is a very long list of covariables and we believe it will make the text harder for the reader to interpret | Line 272 |
| 53 | The second part of this ‘meaning that…’ goes in the discussion | We believe it is important to have a clarification of meaning for the reader, instead of having this in the discussion and refer to this part. This is a literal presentation of the results without further interpretation | Line 273 |
| 54 | 3.3 Care giver perception Lines 253-255 do not belong in results | this is just a remind to keep track of what we did. We believe it helps to interpret the study. | Line 285-287 |
| 55 | Line 255- again which kind of longitudinal model?. Consider splitting into two sentences with the lack of interaction it own sentence | Modified on the new version. | Line 287 |
| 56 | Line 258- don’t need the introductory phrase ‘concerning the care giber’s perception of the scratching problem | This has been suppressed | Line 291 |
| 57 | Line 259-260 I think you mean report? Is there a Chi squared and p value to go with this? | Added in the new version | Line 292 |
| 58 | Line 261-262: what kind of correlation? R squared, rho? Please report the p value and regression type for this correlation | R squared added directly in the graph and in the text of the new version with p-value. | Line 291-295 |
| 59 | Line 265- this is the first time disturbingness is mentioned that it’s limited to prior 7 days, please add this to the method section. Shortened line 266 to “evaluation of scratching over last 28 days using chi squared | The mentioned of disturbing is present in the methods we clarify the fact that this question was asked every week. The line has been shortened. | Line 149 |
| 60 | Line 270- please defined VeDDRA as this is it’s first time being mentioned | This has been changed | Line 304 |
| 61 | Line 274- please include in the method section that adverse events were recorded | Added in the Method section and clarification in this section | Line 124 and line 302-303 |
| 62 | Please report a p value for the comparison between pheromone and placebo adverse events. There are again no asterisks in the associated figure | The p values were not calculated because the percentages were for most of them higher for placebo. | See Figure 5 |
| 63 | Line 277- this interpretive sentence goes in the discussion | Changed as suggested. | Line 383 |
| 64 | Line 288- consider removing ‘only’ and ‘on’ for improved clarity and flow | This was changed | Line 320 |
| 65 | Line 289- introduce the idea of safety signals earlier or define here | It is mentioned in the Introduction. Changed “signals” to “messages” to be more in line with what is written in the introduction | Line 43 – L47 |
| 66 | This is the first time you have used the abbreviation FFP, please introduce earlier or define or both | Abbreviation FFP was present in introduction | Line 85 |
| 67 | Line291-292- how is at home with clients a controlled setting? Do you mean placebo controlled? | Yes, we changed this | Line 323 |
| 68 | Line 292- consider ‘in keeping with’ instead of “is not surprising considering” | Changed as suggested. | Line 324 |
| 69 | Line 294- remove ‘that’ for easier reading | Changed as suggested. | Line 328 |
| 70 | Line 296: remove ‘of cats for easier reading. Would like a citation for this statement | Changed as suggested. | Line 328 |
| 71 | Line 297- ‘considering that’ is unnecessary, removing ‘in this study’ would increase ease of reading | This was changed | Line 329 |
| 72 | would be best to compare to the placebo group and end the sentence at source. Separate line 300 on into it’s own sentence or use a semi colon | Changed as suggested. | Line 331-332 |
| 73 | Line 301- I contend that if you have to lump improved with stopped completely this is a ‘promising’ product, not a ‘highly promising’ product | Changed as suggested | Line 332 |
| 74 | Line 303 “ however’ will be shorter to ready than ‘on the other hand’ you also did not quite present an ‘on the one hand’ | This has been changed | Line 335 |
| 75 | Line 305- please define what ‘’correct use’ of scratching post means here- do you mean providing appropriate posts in appropriate location by the client, or do you mean scratching the provided posts by the cat? | This was clarified | Line 337-338 |
| 76 | Line 308- sub ‘had’ for were reported to have’ for ease of reading. | This has been changed | Line 340 |
| 77 | Line 310- had, (past tense not have) | This has been changed | Line 342 |
| 78 | Line 311- might be clearer to say taller or longer and length vs size of the cat. | This has been changed | Line 343 |
| 79 | Line 312-316 do you mean your study, or the one that the previously cited one (4,5,720). If this is from you study, you need to include information about the cats scratching posts from the recruitment phase in your methods and in your results if you want to include it in your discussion | Added on the methods | Line 160 |
| 80 | Please also convert all to past tense. “more than half the cats that participated in the study did not seme to” is redundant to the percentage presented above. This sentence also is too long and should be reworked into multiple sentences | This has been changed |  |
| 81 | Line 318- consider starting at Even though. What about location of post? | This has been changed. The percentage are available in S1 Table | Line 349 and S1 Table |
| 82 | Line 321-322- consider reported vs declared, and ‘based on memory’ and ‘after potentially observing the cat more closely because of the study” for better clarity. | This has been changed | Line 352 |
| 83 | Line 324- the parenthetical is unnecessary | This has been changed | Line 355 |
| 84 | Line 326- double check with editor if personal pronouns are allowed often they are not | This has been changed | Line 356 |
| 85 | Line 328- use past tense | This has been changed | Line 359 |
| 86 | Line 329-330- split into two sentences for easier reading | This has been changed | Line 361 |
| 87 | Line 332- this sentence actually seems unrelated to the rest of the paragraph- please move somewhere else or remove entirely | We believe all of them make sense as we are talking about safety - if a cat is fearful of strangers, we are talking about the cat feeling unsafe/not relaxed |  |
| 88 | Line 335: consider ‘caregivers reported cats had completely stopped scratching in contrast to the placebo group (7.2%).’ | This has been changed | Line 367 |
| 89 | Line 338- remove ‘was still considered with a’ and place ‘was still’ after the word ‘mean’ | This has been changed | Line 369 |
| 90 | Line 339- please use suggests or indicates instead of ‘means’ since Mean was just used to refer to a number. Consider reworking this sentence to explain that previous memories influence reporting on disturbingness vs calling it a residual. | This has been changed | Line 370 |
| 91 | Line 341- easier to read as ‘it is interesting to note the difference…” | This has been changed | Line 372 |
| 92 | Line 345- convert to past tense, do you mean could not have been counted by the owner? | Yes, This has been changed | Line 375-377 |
| 93 | Line 348- this is a REALLY high number of adverse events for a pheromone based on clinical experience and generally held belief that you cannot overdose on this product. Please report and discuss how this compares to other Feliway Studies. Please also discuss nocebo and placebo effects | It is important to remind that the owners were asked weekly to report any change on their cats even if not related to product application (see caption of figure 5). This might explain the apparently high number of adverse events. Also, most of them were under the option “change in behavior” observed during the previous week and been classified as “behaviour disorder”. This could be for example “was sleeping more” which might simply happen because of hot day summer. | Line 379-381 |
| 94 | Line 363- shorten to “Feliway Classic Diffuser reduces frequency and intensity of undesired scratching”. | This has been changed | Line 397 |
| 95 | Lines 365- 367- the stress derivation of scratching was your a priori assumption, and was not tested here so you can’t say this paper supports cats scratching furniture may be in poor welfare state. Rework this please | This has been changed | Line 399-403 |
| 96 | Line 367- start the sentence at ‘the use of this simple and innocuous’ | This has been changed | Line 404 |
| 97 | Also please discuss the side effects rates in other papers before declaring this to be innocuous. Line 369= be explicit about unpleasant procedures- nail caps? Onchyectomy? This won’t decrease the need for say dental cleanings which may also be unpleasant for cats | This has been changed | Line 406 |
| 98 | FIGURE1- the ‘recruiting phase’ is referenced several times in the paper but does not appear in the diagram. | The selection questionnaire is clearly identified in the figure |  |
| 99 | The font condensed (bold with small spaces between letters) and does not read well if printed full page- it looks like the text was converted to image for the red and blue text, consider revising- also please double check if this color scheme is red-green color blind compatible. I did not want to upload the images to an online tester to breach confidentiality | The poor quality of the figure might come from the pdf conversion. We did not see that problem on the original figure. We also couldn’t find how to test easily this figure for color blindness, but we printed it in black and white and it’s readable. All figure have been used tested with Preflight Analysis and Conversion Engine (PACE) digital diagnostic tool |  |
| 100 | FIGURE2- B: instead of calling it efficacy parameters which doesn’t inherently mean anything, can the y axis be titled ‘scratching parameters’? | This has been changed |  |
| 101 | C: can’t read the 3s over the dark purple very well- be consistent, the other graph showing numbers has them above the barks. | This has been changed |  |
| 102 | D: very hard to read the grey over the purple= can this be expanded? | On the Figure this is the maximum, this figure was a challenge and we try the maximum between readability and space. We know this is not ideal, but we did not figure better way, it remain possible to zoom in the figure |  |
| 103 | FIGURE3: all: Please use different indicators for the points such as a dot and a square or a dot and a diamond vs only relying on the color | This has been changed |  |
| 104 | A: Please make the error bars different color or line thickness form the placebo line- why are they're no error bars on day 28? | the error bars are still present but =0, the figure has been change with a slight sift for better readibility |  |
| 105 | C: is this the global score index? Be consistent in your variable names. Again please also use different dogs | Yes this is the global index, we have only one score call index in the all paper. |  |
| 106 | FIGURE 4: could call this ‘Disturbing rating’ for ease of reading and shorting in the axis labels. {Lease use different indictor shapes for the points for each group. | This has been changed |  |
| 107 | FIGURE 5: no asterisks suggests these are all statistically the same rate between placebo and Pheromone. | The p values were not calculated because the percentages were higher for placebo. |  |
| 108 | TABLE 2. This table is exceedingly hard to read. Please revise with each variable on one line in one column, then a row of the levels in that variable to it’s right: Age 7m-2 year 3-6 year 7-10 year Lifestyle Etc. | This has been changed |  |
| 109 | TABLE 4: replace all ‘of’s with ‘by‘ | This has been changed |  |
| 110 | SUPPLEMENTARY MATERAIL: Please use different point indicator for placebo vs pheromone. Fig 1: no asterisks, so these are all not statistically significant? | We did not perform a statistical test because it doesn’t make sense to compare those means. We will end up comparing means obtained with one, 2 or 4 values (that is why the N are clearly detailed). This is not the goal of this figure. We just wanted to highlight the difference observed in the disturbing rate, frequency of scratching and global evaluation. |  |
