# Supplementary material for: Efficacy of the Feliway® Classic Diffuser in reducing undesirable scratching in cats: A randomised, triple-blind, placebo-controlled study
Source: PLoS One. 2023 Oct 18;18(10):e0292188. doi: 10.1371/journal.pone.0292188 (PMC10584138; doi:10.1371/journal.pone.0292188)
Supplement: Attachment — Submitted filename: Response to Reviewers 2.docx [file pone.0292188.s008.docx]

Dear Dr Brundage,

Thank you for letting us the opportunity to publish in PlosOne. We performed changes to meet PlosOne’s publication criteria and answer to the reviewers’ comments.

Following in blue you could find our answers to all points raised by the reviewers.

Reviewer #2:

A few edits for minor things. There is still a set of conclusions in the discussion that does not match the figures. It may be that the figures to be edited to match them as this is what the average reader will check first rather than the re-read the text of results. (when printing the figure on global evaluation of scratching at d28 which is all blue- has no figure label my apologies. This figure has no asterisk denoting significance. I would be surprised if 0 vs 199 was not significantly different distribution for "no change" and it seems like significant differences are discussed in the results as well?

Regarding the supplementary figure (Sup Fig3), it's important to note that we didn't conduct any statistical tests to compare the mean disturbing levels. In the x-axis, we've displayed the disturbing levels rather than their distribution.

The category "no change" serves to emphasize that at Day 28, there were no participants (N=0) who reported "no change" while simultaneously scoring a frequency of 0. In contrast, there were 199 participants who reported "No change" and scored a frequency greater than 0, resulting in a mean disturbing level of approximately 6.

The purpose of this figure was simply to highlight differences observed in disturbing rates, scratching frequency, and overall evaluations. We don't believe that comparing the mean disturbing level within this group would be pertinent to this paper. While we do discuss this figure in the discussion section, we don't mention any statistically significant differences there. Instead, we use this figure to explore and explain the questionnaire, rather than to identify differences between the products used.

We have updated the Figure 4 B to make all corresponding asterisk in the figure and the text.

| **No.** | **Comment/Suggestion** | **Response** | **Line** |
| --- | --- | --- | --- |
| 1 | Line 51: un capitalize Frequency, since you are not yet discussing your data variable. | This has been changed |  |
| 2 | Line 67: replace the commas with decimal points in 0,3 ad 0,9m | This has been changed |  |
| 3 | Line 70: would prefer to have only and (vs and/or), as the implications that cats need more than 1 scratcher, which is typically the case | This has been changed | Line 70 |
| 4 | Line 73-76: may still benefit from being broken up into two sentences. | This has been changed |  |
| 5 | Line 77: again not discussing your data set so decapitalized Frequency | This has been changed |  |
| 6 | Line 97: rephrase with the use of the word “selection” only once | This has been changed |  |
| 7 | Line 115: change to reminder, insert ‘out’ after fill | This has been changed |  |
| 9 | Line 128- the word ‘on’ is erroneous | This has been changed |  |
| 10 | Table 1- If one questionnaire was not answered | This has been changed |  |
| 11 | Line 194: how was “other cats at home” included in the model if only single cat households were used? | This is a residual mistake from another study, we did not have this variable in the model since we only have single cat household | Line 198 |
| 12 | Line 206: can we expand on what ‘owner’s family situation’ means by referencing Table 2? (I first went digging in the supplementary material. | This has been changed | Line 199 |
| 13 | Line 296: for consistency consider capitalizing Disturbing as a variable through out the paper | This has been changed | Line 288 |
| 14 | Line 337: this sentence is a little misleading compared to the figures and stats. Most clients in both groups reported improvement and it was only significantly different in the scratching stopped group per figure 4 B. There are no asterisk indicating significant differences for frequency in Figure 3A… | For more clarity we added all asterisks on the Figure 4B from the Xi 2 test.  It's worth noting that there are no asterisks in the figure 3A because statistical tests couldn't be conducted with the median data as presented. The results of the model with asterisks are available in Supplementary Figure 1. | Line 329 and 332 |
| 15 | Line 349 and 350: replace comma sin your percentages with decimal points | This has been changed | Line 341-342 |
| 16 | Line 410: consider “majority of cats were reportedly unfriendly and hesitate about approaching strangers//” for easier reading. | This has been changed | Line 400 |
